# Back-translating GWAS findings to animal models reveals a role for *Hgfac* and *Slc39a8* in alcohol and nicotine consumption

**DOI:** 10.1038/s41598-022-13283-1

**Published:** 2022-06-04

**Authors:** F. K. El Banna, J. M. Otto, S. M. Mulloy, W. Tsai, S. M. McElroy, A. L. Wong, G. Cutts, S. I. Vrieze, A. M. Lee

**Affiliations:** 1 Department of Pharmacology, University of Minnesota, Minneapolis, MN USA; 2 Department of Psychology, University of Minnesota, Minneapolis, MN USA

**Keywords:** Genetics, Neuroscience, Systems biology

## Abstract

Alcohol and tobacco are the most commonly used addictive substances, with high comorbidity rates between alcohol use disorder and tobacco use disorder. Risk for alcohol and nicotine addiction is highly heritable, and they share common genetic factors. A GWAS in over 1 million individuals has revealed 566 genetic variants in 406 loci associated with multiple stages of alcohol and tobacco use. Three novel genes—*SLC39A8, GRK4 and HGFAC*—within loci associated with altered alcoholic drinks per week (ADW) or cigarettes per day (CPD) were selected to further study their role in alcohol and tobacco use disorder. The role of these genes was assessed using the two-bottle choice addiction paradigm in transgenic mice for each of the genes. We found significant decreases in chronic alcohol consumption and preference in female *Hgfac* knockout (KO) mice, and decreased nicotine preference in male *Hgfac* KO compared with wild-type (WT) mice. Additionally, male *Slc39a8* hypomorph mice showed greater overall nicotine preference compared with WT mice, while no differences were detected for *Grk4* KO mice in alcohol or nicotine consumption and preference in either sex. Thus, this study implicates *Hgfac* and *Slc39a8* in alcohol and tobacco use in a sex-specific manner.

## Introduction

Alcohol and nicotine are the most commonly misused substances, both in the U.S. and worldwide^1,2^. In 2019, 27.8% of adults 21 and older used alcohol products, and 69.5% of adults 18 and older used tobacco products in the past year in the U.S. In addition, over 14.5 million individuals aged 12 and older met criteria for an alcohol use disorder (AUD), and nearly 26 million individuals reported nicotine dependence in 2019, respectively^3^. Moreover, alcohol and tobacco use is highly comorbid, and epidemiological studies indicate that a large proportion of individuals who meet criteria for either an AUD or tobacco use disorder (TUD) also meet criteria for both^4–6^. These traits are highly heterogenous, and numerous complex factors contribute to increased AUD and TUD liability, including genetic and environmental influences. Heritability estimates have shown that genetic factors contribute to approximately 40–60% of the population variability in developing an AUD or TUD, a proportion of which is also common to both disorders^7–10^. However, many of the unique and shared genetic factors remain unknown and understudied. Identifying novel genetic factors can provide a better understanding of the molecular and neural mechanisms that contribute to AUD and/or TUD, and potentially identify targets for the development of pharmacotherapies.

One approach for identifying novel variants and gene loci associated with substance use behaviors is the application of genome-wide association studies (GWAS), which perform agnostic tests of association between a phenotype and common genetic variation across the genome. In recent years, GWAS sample sizes have become sufficiently well-powered to detect robust and replicable loci for substance use disorders (SUDs) and related traits. These GWAS findings also demonstrate that alcohol and tobacco use are highly polygenic, composed of a large number of implicated variants and loci, each with small effect. Nonetheless, large-scale GWAS of alcohol and tobacco use phenotypes to date have yielded significant associations for variants and loci in genes with clear biological relevance for addiction, such as those involved in substance metabolism, neural targets, and dopaminergic neurotransmission (for recent reviews of notable SUD GWAS findings, see^9,11,12^). Many genetic loci that are identified are novel targets for AUD and/or TUD related biology, representing untapped potential in further understanding addiction biology and for developing novel therapies.

Our recent work investigating genetic associations in over one million individuals discovered numerous novel genetic loci and genes that are associated with alcohol and tobacco use^13^. How these novel genes contribute to the neurobiology of AUD and/or TUD has yet to be determined. The objective of this study was to provide pre-clinical behavioral data on novel genes that were associated with alcoholic drinks per week (ADW) or cigarettes smoked per day (CPD) that were identified in the GWAS study^13^, using a mouse model of alcohol and nicotine consumption. We chose genes closest to the target genetic loci where the single nucleotide polymorphism (SNP) predicted a non-synonymous mutation, and which had not been widely investigated in the context of AUD and/or TUD in humans: *HGFAC*, *SLC39A8*, and *GRK4*. *HGFAC* and *GRK4* had not been previously associated with AUD and/or TUD in humans, whereas *SLC39A8* is highly pleiotropic and has previously been associated with AUD^14,15^. *HGFAC* encodes hepatocyte growth factor activator, which is a coagulation factor XII-like serine endopeptidase^16^ and was associated with ADW^13^. *SLC39A8* encodes for Zrt- and Irt-like protein 8 (ZIP8), a zinc transporter^17,18^ and was also associated with ADW^13^. Lastly, *GRK4* encodes for G-protein coupled receptor kinase 4^19^ and was associated with CPD^13^. To observe a potential maximal effect of these genes, we used transgenic mouse lines where the protein products of these genes were deleted or function was severely disrupted. We obtained and tested *Hgfac* global knock-out (KO) mice, *Grk4* global KO mice, and *Slc39a8* heterozygote (HET) hypomorph mice (homozygote *Slc39a8* gene disruption is lethal)^20^ and assessed voluntary, alcohol and nicotine consumption in 2-bottle choice tests^13^. The *HGFAC* and *SLC39A8* variants were associated with reduced ADW in humans^13^, thus we hypothesized that disruption of *Hgfac* and *Slc39a8* would reduce alcohol consumption and preference in mice. The *GRK4* variant was associated with decreased CPD in humans^13^, thus we hypothesized that disruption of *Grk4* would reduce nicotine consumption and preference in mice. We found significant decreases in chronic alcohol consumption and preference in female *Hgfac* KO mice, and differences in nicotine preference in male *Hgfac* KO mice compared with wild-type littermates. There were no changes in nicotine consumption in *Grk4* KO mice. Male *Slc39a8* hypomorph mice showed no changes in alcohol consumption or preference, and an increase in nicotine preference.

## Methods

### Animals and drugs

Adult male and female mice used in all experiments were a minimum of 8 weeks old. The mice began experiments between 8 and 13 weeks old and all cohorts of mice consisted of age-matched littermate pairs. The *Grk4* global KO transgenic breeder mice were obtained from The Jackson Laboratory. The *Hgfac* global KO breeders were a generous gift from Professor Kataoka at Miyazaki University, Japan. The *Slc39a8* HET hypomorph breeders were a generous gift from Dr. Daniel Nebert at the University of Cincinnati and Dr. Zijuan Liu at Oakland University, Michigan, USA. Homozygote *Slc39a8* hypomorph mice are estimated to have 10–15% of wild-type protein levels and are not viable^20^; therefore, all experiments were conducted with *Slc39a8* HET and WT mice. All three transgenic lines were maintained on an C57BL/6 genetic background and bred in-house at the University of Minnesota. For each transgenic line, we investigated behaviors in male and female transgenic mice (KO mice for the *Hgfac* and *Grk4* lines, HET mice for the *Slc39a8* line) and in the WT littermate controls for each of the three lines. All mice were group housed with a maximum of 4 males and maximum of 5 females in a cage under a standard 12-h light/dark cycle until the start of experiments. Mice were individually housed during the voluntary consumption experiments. All animal procedures and experiments were approved by the Institutional Animal Care and Use Committee (IACUC) at the University of Minnesota, and were in accordance with the recommendations of the ARRIVE guidelines^21^.

Alcohol (Decon Labs, King of Prussia, PA), and nicotine tartrate salt (Acros Organics, Thermo Fisher Scientific, Chicago, IL) were mixed with tap water to the concentrations reported for each experiment. The concentrations of nicotine described herein are reported as free base. Nicotine solutions were not filtered or pH adjusted, and neither the alcohol nor nicotine solutions contained sweetener. Saccharine and quinine (MilliporeSigma, Burlington, MA) were mixed with tap water to the concentrations reported for the taste experiments.

### Alcohol two-bottle choice

Drug naïve male and female mice from the three transgenic lines were singly housed in double grommet cages and underwent a continuous access alcohol two-bottle choice procedure that we and other groups have previously used to evaluate alcohol consumption in mice^22–25^. The two-bottle choice procedure was performed as described in our past work investigating voluntary alcohol consumption in mice^22,23^. Briefly, the procedure involves presenting mice with a bottle of water and a bottle containing alcohol in their home cage. The following escalating alcohol concentrations were presented for 4 days each: 3, 6, 10, 14, and 20% v/v in water. Bottle positions were switched every 2 days to account for side preferences. Bottles were weighed every 2 days and mice were weighed each week. Mice had ad libitum access to food and water during the procedure.

### Nicotine two-bottle choice

Drug naïve WT, KO or HET male and female mice were singly housed in double grommet cages and underwent a continuous access nicotine two-bottle choice procedure^22,26,27^. Mice were presented with a bottle of water and a bottle containing nicotine dissolved in water. The nicotine concentration was presented at the following concentrations for 1 week each: 30, 50, 75, and 100 µg/mL. The bottles were weighed every 2–3 days and replaced with fresh solutions every 3–4 days. The positions of the bottles were switched at every weighing to account for side preferences. The mice were weighed each week. Mice had ad libitum access to food and water during the procedure.

### Taste preference test

To determine whether the genes of interest influenced taste preference, and thus could confound the interpretation of the drug 2-bottle choice tests, we assessed the preference for sweet and bitter tasting substances. The taste preference tests were performed in a similar manner as our past work examining sweet and bitter tastes in transgenic mice^23^. In this study, a subset of mice that completed the alcohol or nicotine two-bottle choice tests were given water for a week prior to beginning the taste test. Briefly, the procedure involves a saccharine two-bottle choice test followed by a quinine two-bottle choice with 3 days of water only in between the tests. The mice were presented with one bottle of water and one bottle of saccharine or quinine in their home cage. The saccharine concentrations presented were 1.5 and 15 mM, and the quinine concentrations presented were 0.01 and 0.1 mM. Each concentration was presented for 2 days each and the bottle positions switched after every day to account for side preferences. Mice were weighed each week. Mice had ad libitum access to food and water during the procedure.

### Statistical analysis

Drug consumption (either g/kg/day for alcohol or mg/kg/day for nicotine) was calculated using the weights of the fluid consumed, the concentration and density (for alcohol only) of the solutions and the weights of the mouse. The percent preference for the bottle of interest was calculated as the amount of fluid (g) consumed from the bottle of interest divided by overall fluid consumed from all available bottles multiplied by 100. The consumption and preference were calculated using 2-way repeated measures ANOVA followed by appropriate post-hoc multiple comparison tests using Prism 6.0 (GraphPad, La Jolla, CA). If sex differences in drug consumption were observed in the WT mice, male and female mice were analyzed separately (for each transgenic line), as in past studies^22,24,28^.

## Results

### Hgfac—alcohol consumption and preference

As *HGFAC* was associated with ADW in the GWAS, we first assessed alcohol consumption and preference using a chronic 2-bottle choice test. The *Hgfac* transgenic line is maintained on a C57BL/6 background, and this genetic background has a well-documented sex difference, in which female mice have greater alcohol consumption and preference compared with male mice^22,25,29–31^. We first compared alcohol consumption between male and female *Hgfac* WT mice and found a significant interaction between sex and alcohol concentration (F_interaction_(4,300) = 15.71, *P* < 0.0001, F_concentration_(4,300) = 90.69, *P* < 0.0001, F_sex_(1,75) = 82.91, *P* < 0.0001). Sidak’s multiple comparison testing showed that female mice consumed significantly more alcohol compared with male mice at all concentrations except for 3% (*P* < 0.05 for 6, 10, 14 and 20% between sexes). For alcohol preference, we also found a significant interaction between sex and alcohol concentration (F_interaction_(4,292) = 2.445, *P* = 0.047, F_concentration_(4,292) = 59.90, *P* < 0.0001, F_sex_(1,73) = 22.02, *P* < 0.0001). Sidak’s multiple comparisons testing showed that female mice also had higher preference for alcohol at all concentrations except for 3% (*P* < 0.05 for 6, 10, 14 and 20% between sexes), similar to the results observed for alcohol consumption. Given the observed sex difference in alcohol consumption and preference, we examined the effects of *Hgfac* separately by sex as we have done in prior work^22,24,28^.

**Figure 1:**
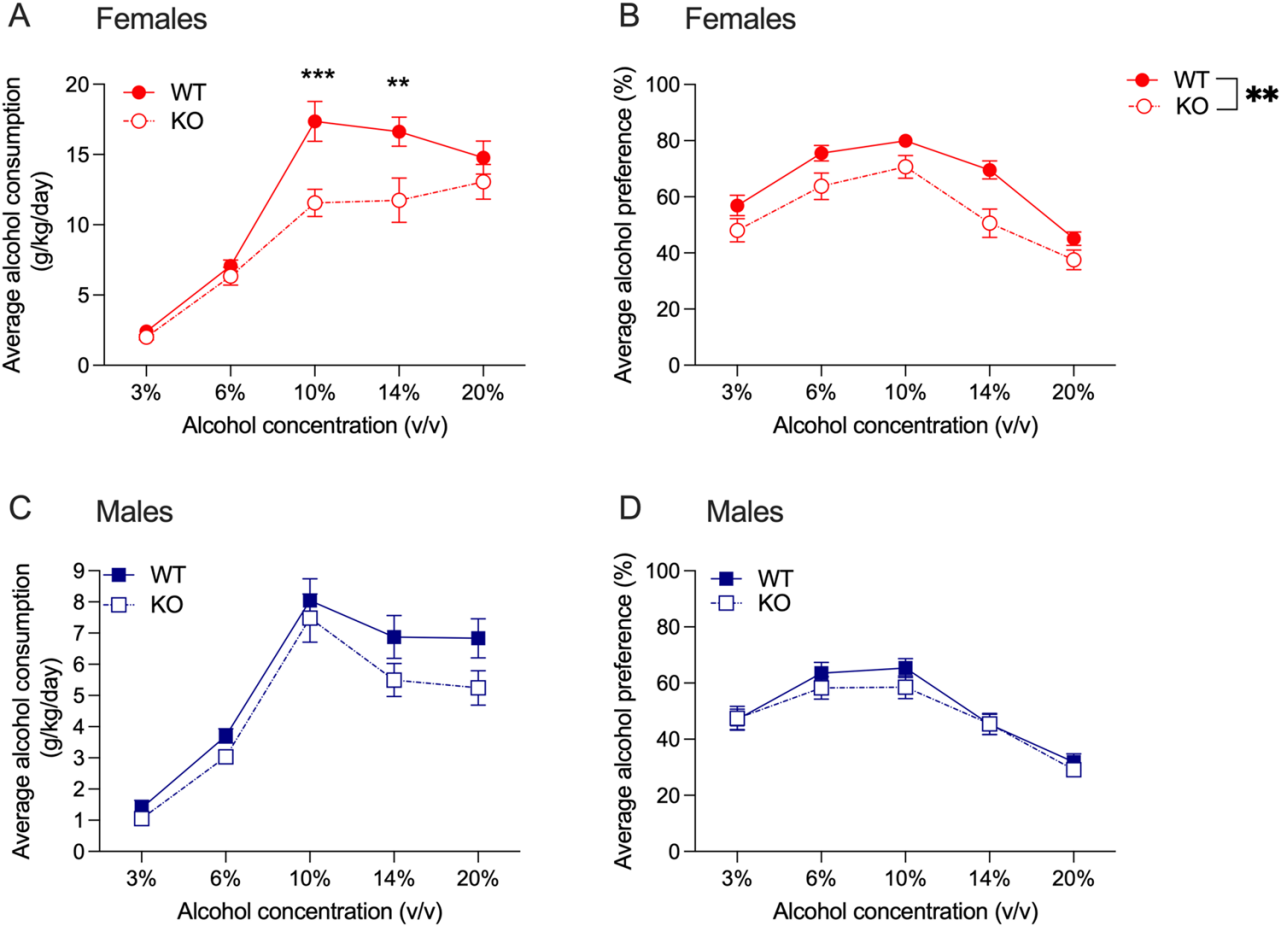
Alcohol consumption and preference in *Hgfac* WT and KO mice. (**A**) Female *Hgfac* KO mice had significantly reduced alcohol consumption at the 10% and 14% alcohol concentrations. Sidak’s multiple comparisons testing **P < 0.01 and ***P < 0.001 compared with WT mice. (**B**) There was a reduction in overall alcohol preference in female *Hgfac* KO mice compared with WT mice. **P = 0.003 for main effect of genotype. There was no difference between (**C**) alcohol consumption and (D) alcohol preference between the male *Hgfac* KO and WT littermates. Data are presented as mean ± SEM, *n* = 28–39 female mice per genotype, *n* = 36–38 male mice per genotype.

For alcohol consumption in female *Hgfac* WT and KO mice, we found a significant interaction between alcohol concentration and genotype (F_interaction_(4,260) = 4.046, *P* = 0.003, F_concentration_(4,260) = 82.19, *P* < 0.0001, F_genotype_(1,65) = 8.473, *P* = 0.005, Fig. 1A). Sidak’s multiple comparisons testing showed that female WT mice consumed more alcohol compared with KO littermates at the 10% and 14% concentrations. For alcohol preference, we found main effects of genotype and alcohol concentration with no interaction (F_interaction_(4, 256) = 1.400, *P* = 0.23, F_concentration_(4, 256) = 49.43, *P* < 0.0001, F_genotype_(1, 64) = 9.804, *P* = 0.003, Fig. 1B). For alcohol consumption in male drug naïve *Hgfac* WT and KO mice, we found a significant main effect of alcohol concentration with no interaction between concentration and genotype, and no main effect of genotype (F_interaction_(4,288) = 0.773, *P* = 0.55, F_concentration_(4,228) = 70.35, *P* < 0.0001, F_genotype_(1,72) = 3.505, *P* = 0.07, Fig. 1C). For alcohol preference in male mice, we found a main effect of concentration, no main effect of genotype and no significant interaction between genotype and concentration (F_interaction_(4,288) = 0.644, *P* = 0.63, F_concentration_(4,228) = 41.57, *P* < 0.0001, F_genotype_(1,72) = 0.621, *P* = 0.43, Fig. 1D). Overall, we found that female *Hgfac* KO mice showed decreased alcohol consumption and preference compared with WT littermates, with no differences observed in male mice.

### Hgfac—Nicotine consumption and preference

We first examined nicotine consumption and preference in *Hgfac* WT males and females to determine whether the documented sex difference in nicotine intake in C57BL/6 mice^22,32,33^ was also present in this C57BL/6-based transgenic line. For nicotine consumption, we found a significant interaction between sex and nicotine concentration (F_interaction_(3,102) = 8.623, *P* < 0.0001, F_concentration_(3,102) = 50.53, *P* < 0.0001, F_sex_(1,34) = 20.86, *P* < 0.0001). Sidak’s multiple comparisons testing showed that female *Hgfac* WT mice consumed more nicotine at the 75 and 100 μg/mL concentrations compared with male *Hgfac* WT mice. For nicotine preference, we found that female *Hgfac* WT mice showed greater overall nicotine preference compared with male *Hgfac* WT mice, with significant main effects of sex and nicotine concentration without an interaction between sex and nicotine concentration (F_interaction_(3,102) = 0.014, *P* = 0.99, F_concentration_(3,102) = 3.469, *P* = 0.02, F_sex_(1,34) = 6.514, *P* = 0.02).

**Figure 2:**
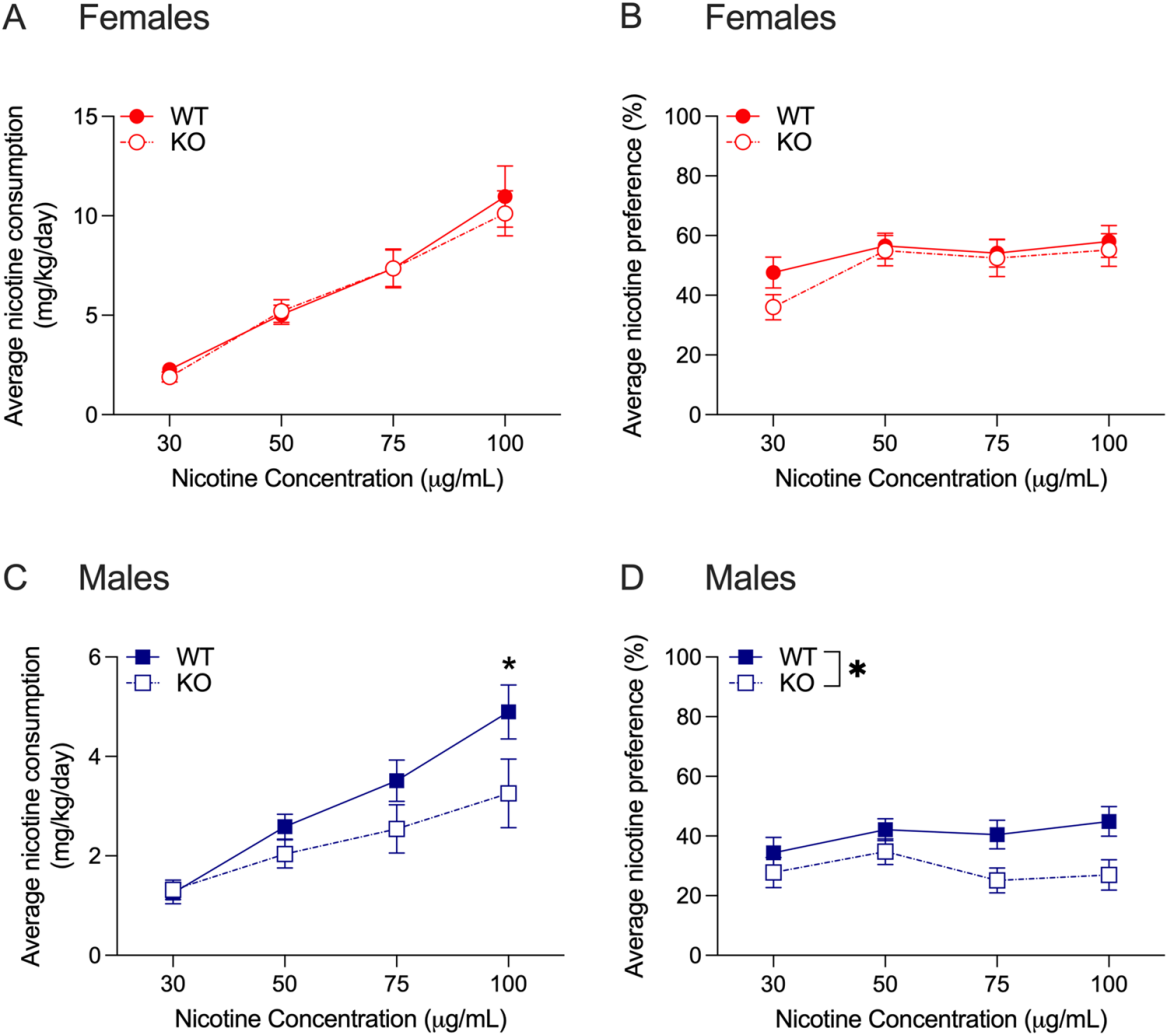
Nicotine consumption and preference in *Hgfac* WT and KO mice. Female *Hgfac* WT and KO mice showed no significant differences in (**A**) nicotine consumption or (**B**) preference. (**C**) Male *Hgfac* KO mice consumed less nicotine at the 100 μg/mL concentration compared with WT littermates. Sidak’s multiple comparisons testing *P < 0.05 between WT and KO mice. (**D**) Male *Hgfac* KO mice consumed less nicotine overall compared with WT mice. *P = 0.04 for main effect of genotype. Data are presented as mean ± SEM, *n* = 17–18 female mice per genotype, *n* = 16–19 male mice per genotype.

In female drug naïve *Hgfac* WT and KO mice, we found a main effect of nicotine concentration with no significant interaction between nicotine concentration and genotype, and no main effect of genotype (F_interaction_(3,99) = 0.275, *P* = 0.84, F_concentration_(3,99) = 68.62, *P* < 0.0001, F_genotype_(1,33) = 0.069, *P* = 0.79, Fig. 2A). For nicotine preference in female mice, we found a main effect of concentration with no interaction between nicotine concentration and genotype, and no main effect of genotype (F_interaction_(3,99) = 1.212, *P* = 0.31, F_concentration_(3,99) = 9.707, *P* < 0.0001, F_genotype_(1,33) = 0.518, *P* = 0.48, Fig. 2B). For nicotine consumption in male drug naïve *Hgfac* WT and KO mice, we found a significant interaction between nicotine concentration and genotype (F_interaction_(3,99) = 3.267, *P* = 0.03, F_concentration_(3,99) = 35.25, *P* < 0.0001, F_genotype_(1,33) = 2.619, *P* = 0.12, Fig. 2C). Sidak’s multiple comparisons test showed that the male WT mice consumed more nicotine at the 100 μg/mL concentration compared with KO littermates. For nicotine preference, we found a significant main effect of genotype without an interaction between nicotine concentration and genotype or a main effect of genotype (F_interaction_(3,99) = 1.857, *P* = 0.14, F_concentration_(3,99) = 2.459, *P* = 0.07, F_genotype_(1,33) = 4.451, *P* = 0.04, Fig. 2D). Overall, we found that male *Hgfac* KO mice showed reduced nicotine consumption and preference compared with WT littermates, with no differences observed in female mice.

### Hgfac—Taste preference

**Figure 3:**
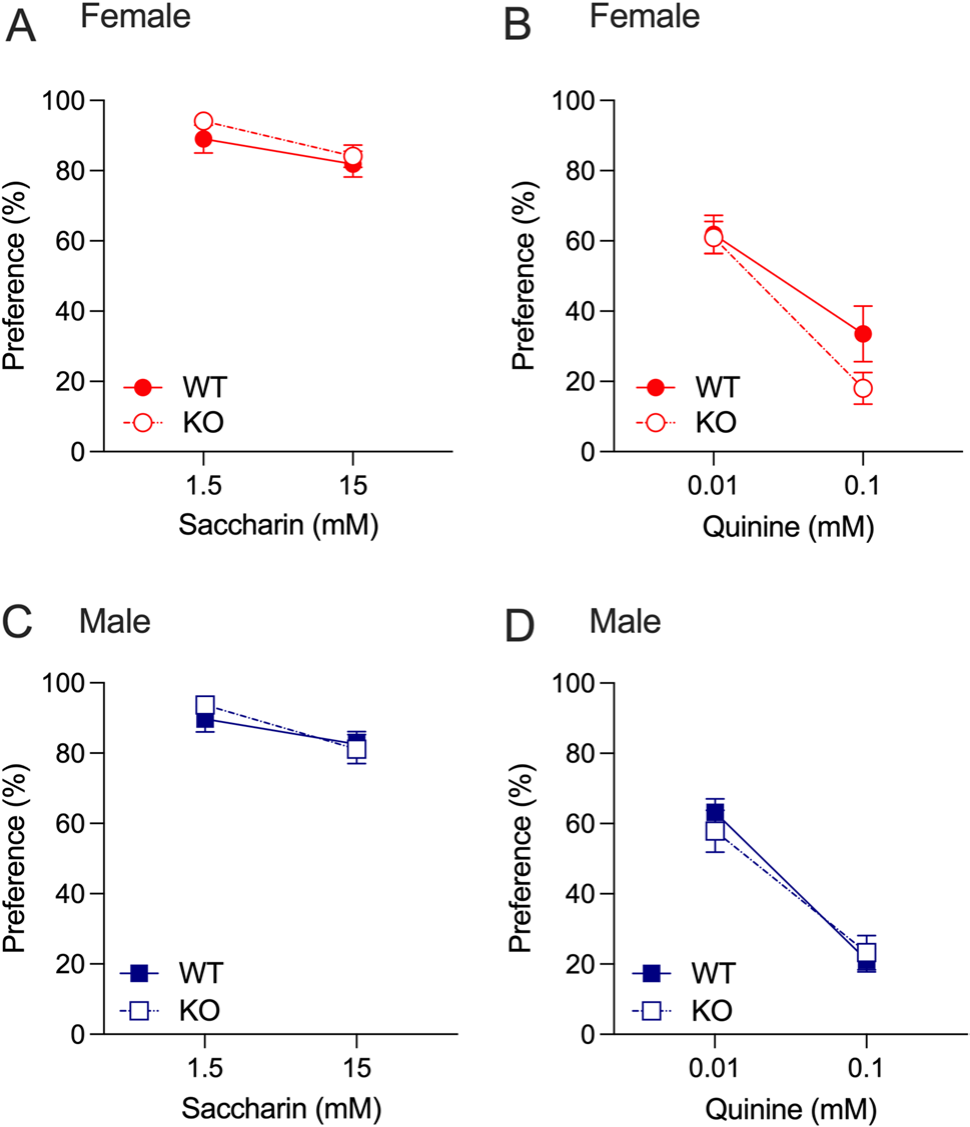
Saccharine and quinine consumption and preference in *Hgfac* WT and KO mice. Female *Hgfac* WT and KO mice showed no significant differences in (**A**) saccharine or (**B**) quinine preference. Male *Hgfac* WT and KO mice also showed no significant differences in (**C**) saccharine or (**D**) quinine preference. Data are presented as mean ± SEM, *n* = 12–13 female mice per genotype, *n* = 13–14 male mice per genotype.

As alcohol and nicotine preference can be influenced by differences in taste preference, we compared saccharine and quinine preference in *Hgfac* WT and KO mice. In female mice, both saccharine and quinine produced a main effect of concentration only with no interaction between concentration and genotype and no main effect of genotype (saccharine: F_interaction_(1,24) = 0.184, *P* = 0.67, F_concentration_(1,24) = 6.396, *P* = 0.02, F_genotype_(1,24) = 1.427, *P* = 0.24, Fig. 3A; quinine: F_interaction_(1,23) = 1.995, *P* = 0.17, F_concentration_(1,23) = 47.39, *P* < 0.0001, F_genotype_(1,23) = 1.589, *P* = 0.22, Fig. 3B). Similar results were observed in the male *Hgfac* WT and KO mice, with a main effect of concentration only for both saccharin and quinine preference (saccharine: F_interaction_(1,25) = 0.479, *P* = 0.50, F_concentration_(1,25) = 6.412, *P* = 0.02, F_genotype_(1,25) = 0.255, *P* = 0.62, Fig. 3C; quinine: F_interaction_(1,25) = 0.784, *P* = 0.38, F_concentration_(1,25) = 82.10, *P* < 0.0001, F_genotype_(1,25) = 0.099, *P* = 0.76, Fig. 3D). Overall, we observed no changes in sweet or bitter taste preference in either sex of *Hgfac* KO mice.

### Slc39a8—Alcohol consumption and preference

*SLC39A8* was associated with ADW in the GWAS, thus we examined 2-bottle choice alcohol consumption. As the *Slc39a8* transgenic line is also maintained on a C57BL/6 background, we first examined alcohol consumption and preference across sex in WT mice. For alcohol consumption, we found a significant interaction between sex and alcohol concentration (F_interaction_(4,232) = 17.48, *P* < 0.0001, F_concentration_(4,232) = 47.47, *P* < 0.0001, F_sex_(1,58) = 45.58, *P* < 0.0001). Sidak’s multiple comparisons testing showed that female *Slc39a8* WT mice consumed more alcohol compared with male mice at all concentrations except for 3% (*P* < 0.05 for 6, 10, 14 and 20% compared with male *Slc39a8* WT mice). For alcohol preference, we also found a significant interaction between sex and alcohol concentration (F_interaction_(4,236) = 2.691, *P* = 0.03, F_concentration_(4,236) = 16.53, *P* < 0.0001, F_sex_(1,59) = 6.027, *P* = 0.02). Sidak’s multiple comparisons testing showed that female *Slc39a8* WT mice had a greater preference for alcohol at the 10 and 14% concentrations compared with male *Slc39a8* WT mice.

**Figure 4:**
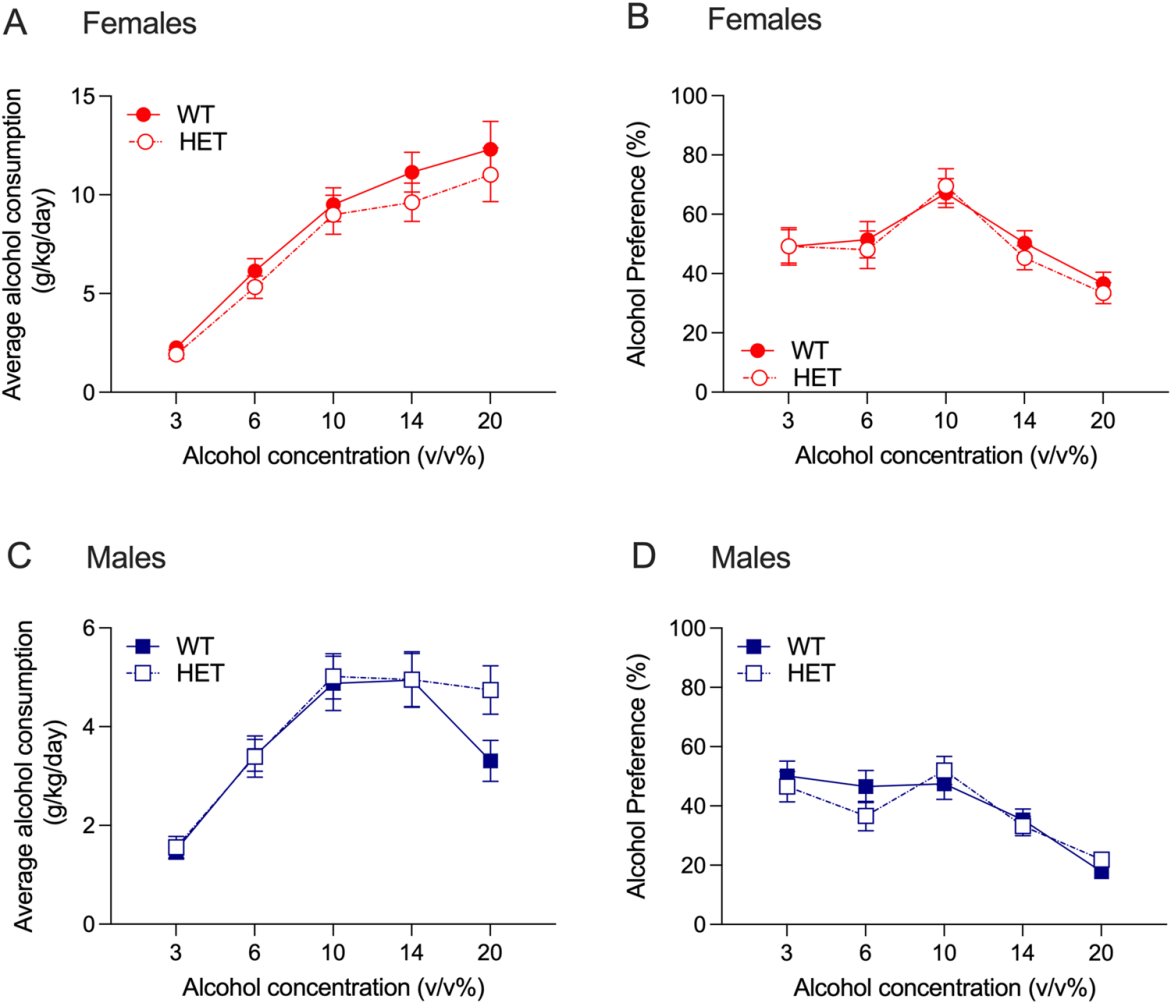
Alcohol consumption and preference in *Slc39a8* WT and HET mice. No differences between *Slc39a8* WT and HET mice were observed for (**A**) female alcohol consumption, (**B**) female alcohol preference, (**C**) male alcohol consumption and (**D**) male alcohol preference. Data are presented as mean ± SEM, *n* = 28 female mice per genotype, *n* = 32–33 male mice per genotype.

We tested chronic 2-bottle choice alcohol consumption and preference in drug naïve *Slc39a8* HET and WT mice. For alcohol consumption in female drug naïve *Slc39a8* HET and WT mice, we found a main effect of alcohol concentration with no interaction between concentration and genotype, and no main effect of genotype (F_interaction_(4,216) = 0.235, *P* = 0.92, F_concentration_(4,216) = 56.01, *P* < 0.0001, F_genotype_(1,54) = 0.998, *P* = 0.32, Fig. 4A). We observed similar results for alcohol preference in the female mice, with a main effect of alcohol concentration only (F_interaction_(4,216) = 0.228, *P* = 0.92, F_concentration_(4,216) = 14.75, *P* < 0.0001, F_genotype_(1,54) = 0.144, *P* = 0.71, Fig. 4B). In the male drug naïve *Slc39a8* HET and WT mice, we also found a significant main effect of alcohol concentration only for both alcohol consumption (Fig. 4C) and preference (Fig. 4D) (alcohol consumption: F_interaction_(4,252) = 1.649, *P* = 0.16, F_concentration_(4,252) = 33.79, *P* < 0.0001, F_genotype_(1,63) = 0.554, *P* = 0.46; alcohol preference: F_interaction_(4,256) = 1.384, *P* = 0.24, F_concentration_(4,256) = 23.10, *P* < 0.0001, F_genotype_(1,64) = 0.1159, *P* = 0.73). Overall, we observed no effect of the *Slc39a8* hypomorph on alcohol consumption or preference in either sex.

### Slc39a8—nicotine consumption and preference

We first examined nicotine consumption in *Slc39a8* WT male and female mice and found an interaction between nicotine concentration and sex (F_interaction_(3,153) = 3.910, *P* = 0.01, F_concentration_(3,153) = 45.77, *P* < 0.0001, F_sex_(1,51) = 14.23, *P* < 0.001). Sidak’s multiple comparisons testing showed that female *Slc39a8* WT mice consumed more nicotine compared with male mice at the 50, 75 and 100 μg/mL concentrations (all *P* < 0.05 between sex). For nicotine preference, we found a main effect of sex such that female *Slc39a8* WT mice had greater overall nicotine preference compared with male mice. There was no interaction between sex and concentration and no main effect of nicotine concentration itself (F_interaction_(3,153) = 0.640, *P* = 0.59, F_concentration_(3,153) = 0.177, *P* = 0.91, F_sex_(1,51) = 6.565, *P* = 0.01).

**Figure 5:**
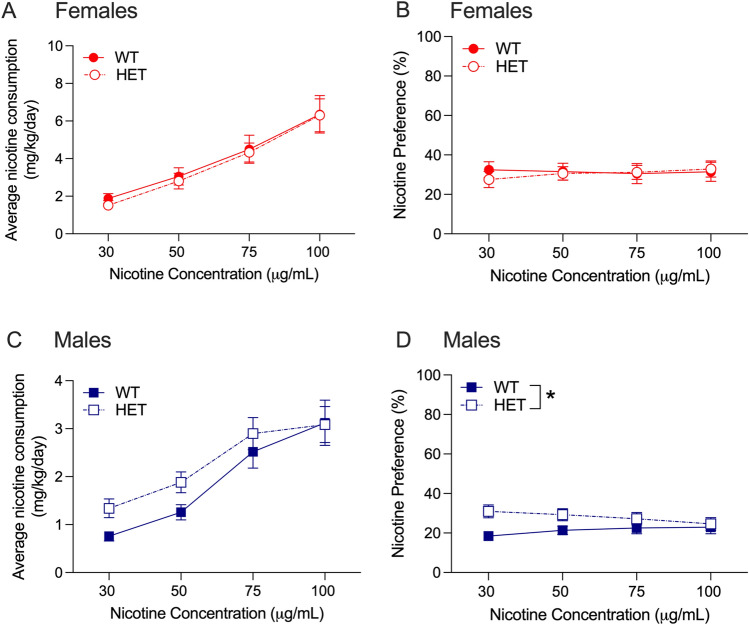
Nicotine consumption and preference in *Slc39a8* WT and HET mice. No differences between female *Slc39a8* WT and HET mice were observed for (**A**) nicotine consumption or (**B**) nicotine preference. (**C**) No genotype differences were observed in nicotine consumption between male *Slc39a8* WT and HET mice. (**D**) Male *Slc39a8* HET mice showed greater overall nicotine preference compared with WT littermates. *P = 0.049 for main effect of genotype. Data are presented as mean ± SEM, *n* = 21–22 female mice per genotype, *n* = 32–38 male mice per genotype.

We tested nicotine consumption in female drug naïve *Slc39a8* WT and HET mice, and found a main effect of nicotine concentration with no interaction between nicotine concentration and genotype, and no main effect of genotype (F_interaction_(3,123) = 0.05, *P* = 0.99, F_concentration_(3,123) = 46.16, *P* < 0.0001, F_genotype_(1,41) = 0.09, *P* = 0.77, Fig. 5A). For nicotine preference in female drug naïve *Slc39a8* WT and HET mice, we found no significant interaction between nicotine concentration and genotype, and no main effects of genotype or nicotine concentration (F_interaction_(3,123) = 0.541, *P* = 0.66, F_concentration_(3,123) = 0.213, *P* = 0.89, F_genotype_(1,41) = 0.039, *P* = 0.85, Fig. 5B). For nicotine consumption in male drug naïve *Slc39a8* WT and HET mice, we found a main effect of nicotine concentration only and no main effect of genotype or an interaction between genotype and concentration (F_interaction_(3,204) = 0.9384, *P* = 0.42, F_concentration_(3,204) = 37.87, *P* < 0.0001, F_genotype_(1,68) = 1.422, *P* = 0.24, Fig. 5C). However, for nicotine preference in male drug naïve *Slc39a8* mice, we found a main effect of genotype such that the *Slc39a8* HET mice showed greater overall nicotine preference compared with WT littermates (Fig. 5D). We also observed a trend for a significant interaction between nicotine concentration and genotype and no main effect of nicotine concentration itself (F_interaction_(3,204) = 2.395, *P* = 0.07, F_concentration_(3,204) = 0.1849, *P* = 0.91, F_genotype_(1,68) = 4.034, *P* = 0.049, Fig. 5D). To further examine drinking behavior, we analyzed the total fluid consumed over the 4-week experiment normalized by body weight (g/kg) in the male *Slc39a8* HET and WT mice. We found a main effect of week and a main effect of genotype, with no interaction between week and genotype (F_interaction_(3,204) = 0.549, *P* = 0.65; F_week_(3,204) = 9.442, *P* < 0.0001; F_genotype_(1,68) = 4.983, *P* = 0.03). Male *Slc39a8* HET mice drank an average of 11.6% less total fluid by body weight compared with male WT littermates throughout the entire experiment. There was also no difference in the average body weight between male *Slc39a8* HET and WT mice (HET: 26.6 ± 0.5 g, WT: 26.3 ± 0.6 g, t = 0.78, *P* = 0.63). Overall, we observed an increase in nicotine preference in male *Slc39a8* HET mice with no effect on nicotine consumption. This increased preference was present despite a small reduction in overall fluid intake in the male *Slc39a8* HET mice. No genotype effects were observed for female mice.

### Slc39a8—taste preference

**Figure 6:**
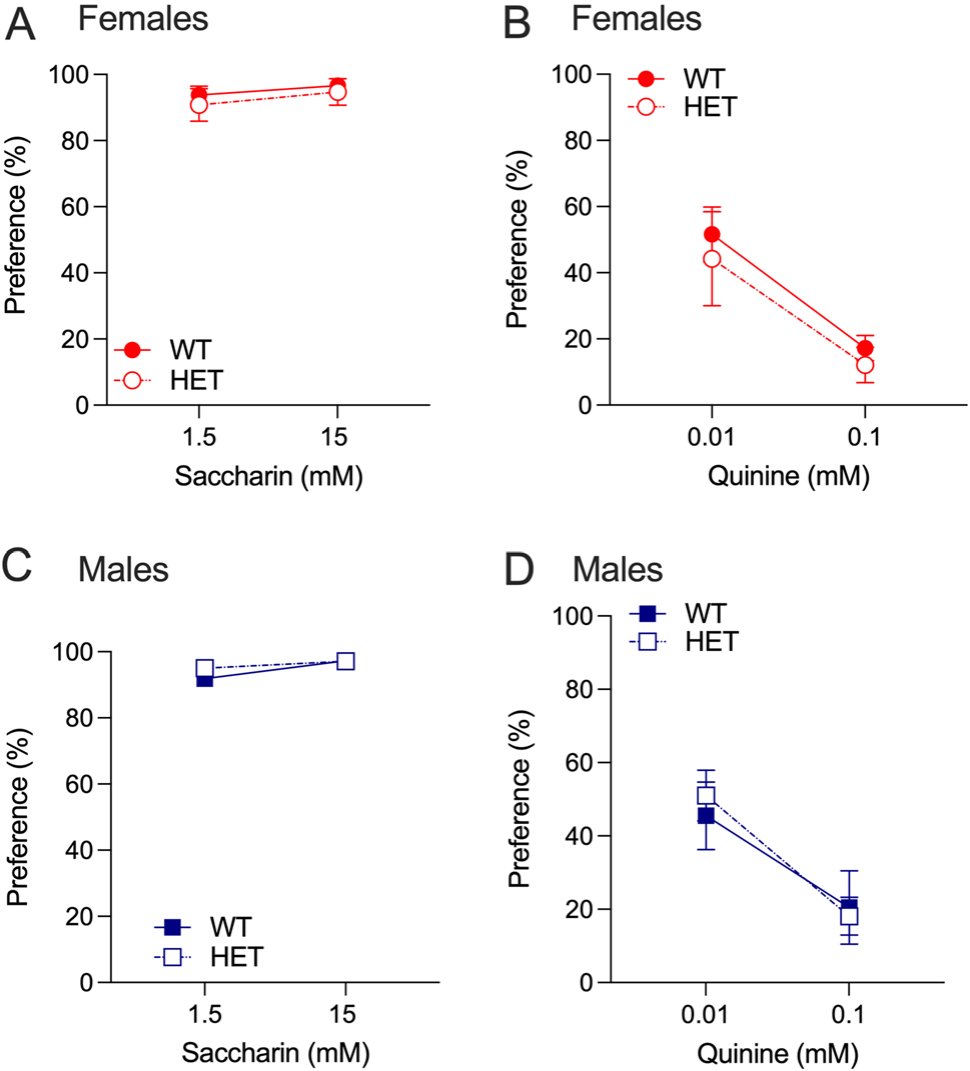
Saccharine and quinine consumption and preference in *Slc39a8* WT and HET mice. Female *Slc39a8* WT and HET mice showed no significant differences in (**A**) saccharine or (**B**) quinine preference. Male *Slc39a8* WT and HET mice also showed no significant differences in (**C**) saccharine or (**D**) quinine preference. Data are presented as mean ± SEM, *n* = 5–8 female mice per genotype, *n* = 7–10 male mice per genotype.

In female *Slc39a8* WT and HET mice, there was no main effect of saccharin concentration or an interaction between saccharin concentration and genotype (F_interaction_(1,11) = 0.043, *P* = 0.84, F_concentration_(1,11) = 1.440, *P* = 0.26, F_genotype_(1,11) = 0.5012, *P* = 0.49, Fig. 6A). Similarly, we found a main effect of quinine concentration without an interaction between quinine concentration and genotype (F_interaction_(1,11) = 0.028, *P* = 0.87, F_concentration_(1,11) = 23.71, *P* < 0.001, F_genotype_(1,11) = 0.432, *P* = 0.52, Fig. 6B). In male *Slc39a8* WT and HET mice, we observed main effects of saccharin and quinine concentration with no main effect of genotype or an interaction between concentration and genotype (saccharine: F_interaction_(1,15) = 1.707, *P* = 0.21, F_concentration_(1,15) = 8.768, *P* = 0.01, F_genotype_(1,15) = 0.9721, *P* = 0.34, Fig. 6C; quinine: F_interaction_(1,15) = 0.3869, *P* = 0.54, F_concentration_(1,15) = 20.74, *P* < 0.001, F_genotype_(1,15) = 0.032, *P* = 0.86, Fig. 6D).

### Grk4—nicotine consumption and preference

*GRK4* was associated with CPD in the GWAS, thus we first assessed nicotine 2-bottle choice. As this transgenic line is also maintained on a C57BL/6 background, we examined sex differences in WT mice. For nicotine consumption, we found a significant interaction between nicotine concentration and sex (F_interaction_(3,93) = 9.959, *P* < 0.0001, F_concentration_(3,93) = 58.20, *P* < 0.0001, F_sex_(1,31) = 18.24, *P* < 0.0001). Sidak’s multiple comparisons testing showed that female *Grk4* WT mice had greater nicotine consumption compared with male mice at the 50, 75 and 100 μg/mL concentrations. For nicotine preference, we found a main effect of sex such that female *Grk4* WT mice had higher overall preference compared with male mice. There was a main effect of nicotine concentration, and no interaction between nicotine concentration and sex (F_interaction_(3,93) = 2.483, *P* = 0.07, F_concentration_(3,93) = 5.707, *P* = 0.0001, F_sex_(1,31) = 4.277, *P* = 0.04).

**Figure 7:**
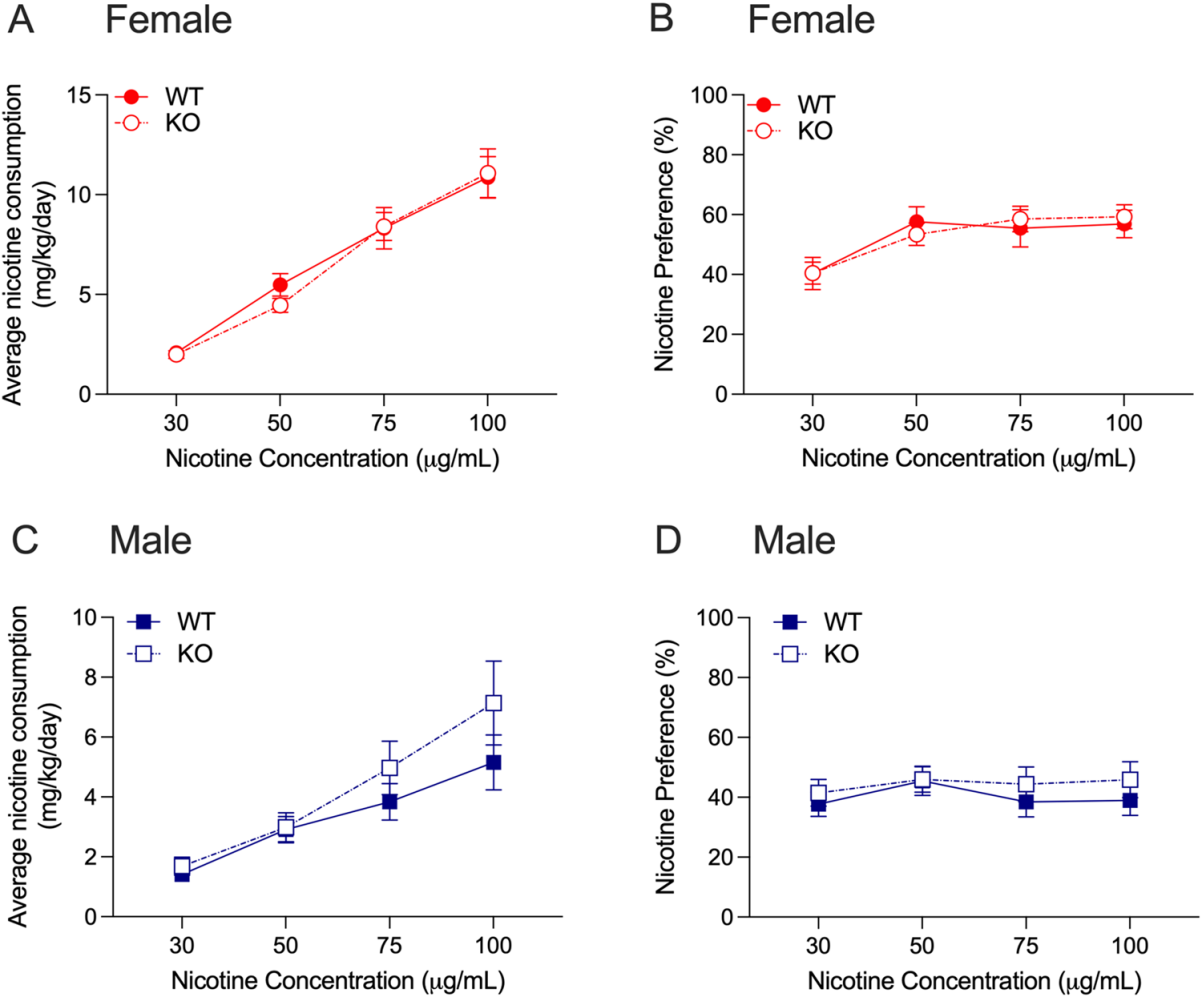
Nicotine consumption and preference in *Grk4* WT and KO mice. No differences between female *Grk4* WT and KO mice were observed for (**A**) nicotine consumption or (**B**) nicotine preference. No genotype differences were observed in (**C**) nicotine consumption or (**D**) nicotine preference between male *Grk4* WT and KO mice. Data are presented as mean ± SEM, *n* = 16–25 female mice per genotype, *n* = 16–17 male mice per genotype.

For nicotine consumption in drug naïve female *Grk4* WT and KO mice, we found a main effect of nicotine concentration, no significant interaction between nicotine concentration and genotype, and no main effect of genotype (F_interaction_(3,117) = 0.436, *P* = 0.73, F_concentration_(3,117) = 84.20, *P* < 0.0001, F_genotype_(1,39) = 0.059, *P* = 0.81, Fig. 7A). For nicotine preference in female mice, we found similar results with a main effect of nicotine concentration, no interaction between nicotine concentration and genotype, and no main effect of genotype (F_interaction_(3,117) = 0.751, *P* = 0.52, F_concentration_(3,117) = 18.82, *P* < 0.0001, F_genotype_(1,39) = 0.004, *P* = 0.95, Fig. 7B). For nicotine consumption in drug naïve male *Grk4* WT and KO mice, we found a main effect of nicotine concentration with no interaction between nicotine concentration and genotype, and no main effect of genotype (F_interaction_(3,93) = 1.720, *P* = 0.17, F_concentration_(3,93) = 34.90, *P* < 0.0001, F_genotype_(1,31) = 0.971, *P* = 0.33, Fig. 7C). For nicotine preference in male mice, we observed no interaction between nicotine concentration and genotype, and no main effects of nicotine concentration or genotype (F_interaction_(3,93) = 0.392, *P* = 0.76, F_concentration_(3,93) = 1.286, *P* = 0.28, F_genotype_(1,31) = 0.5394, *P* = 0.47, Fig. 7D). Overall, we observed no effect of *Grk4* deletion in nicotine consumption or preference in either sex.

### Grk4—alcohol consumption and preference

For alcohol consumption in the *Grk4* WT mice, we found an interaction between alcohol concentration and sex (F_interaction_(4,68) = 5.526, *P* < 0.001, F_concentration_(4,68) = 16.78, *P* < 0.0001, F_sex_(1,17) = 23.98, *P* < 0.001). Sidak’s multiple comparisons testing showed that female *Grk4* WT mice had higher alcohol consumption at the 10, 14 and 20% concentrations compared with male mice. For alcohol preference, we found a main effect of alcohol concentration. There was a non-significant trend for a main effect of sex, and no interaction between sex and alcohol concentration (F_interaction_(4,80) = 0.1416, *P* = 0.97, F_concentration_(4,80) = 42.07, *P* < 0.0001, F_sex_(1,20) = 4.021, *P* = 0.06).

**Figure 8:**
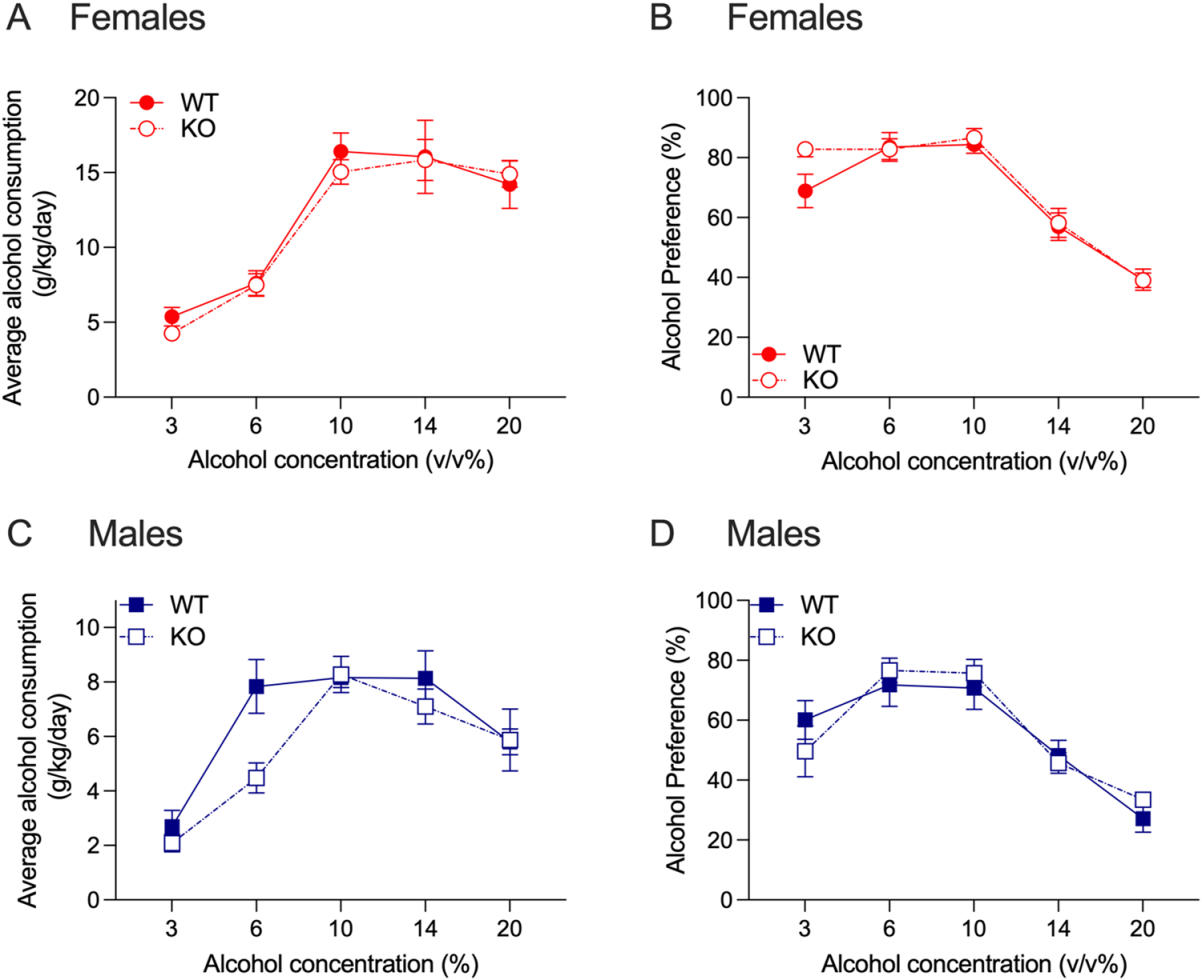
Alcohol consumption and preference in *Grk4* WT and KO mice. No differences between female *Grk4* WT and KO mice were observed for (**A**) alcohol consumption or (**B**) alcohol preference. No genotype differences were observed in (**C**) alcohol consumption or (**D**) alcohol preference between male *Grk4* WT and KO mice. Data are presented as mean ± SEM, *n* = 10–11 female mice per genotype, *n* = 7–11 male mice per genotype.

For alcohol consumption in drug naïve female *Grk4* WT and KO mice, we found a main effect of alcohol concentration with no interaction between alcohol concentration and genotype, and no main effect of genotype (F_interaction_(4,76) = 0.2627, *P* = 0.90, F_concentration_(4,76) = 41.21, *P* < 0.0001, F_genotype_(1,19) = 0.1614, *P* = 0.69, Fig. 8A). For alcohol preference in female mice, we observed similar results with only a main effect of alcohol concentration (F_interaction_(4,76) = 1.644, *P* = 0.17, F_concentration_(4,76) = 69.30, *P* < 0.0001, F_genotype_(1,19) = 0.7833, *P* = 0.39, Fig. 8B). For alcohol consumption in drug naïve male *Grk4* WT and KO mice, we observed a main effect of alcohol concentration with no interaction between alcohol concentration and genotype, and no main effect of genotype (F_interaction_(4,64) = 2.061, *P* = 0.10, F_concentration_(4,64) = 21.05, *P* < 0.0001, F_genotype_(1,16) = 3.234, *P* = 0.09, Fig. 8C). Similarly, for alcohol preference in male mice, we observed a main effect of alcohol concentration only (F_interaction_(4,64) = 0.9941, *P* = 0.42, F_concentration_(4,64) = 26.99, *P* < 0.0001, F_genotype_(1,16) = 0.011, *P* = 0.92, Fig. 8D). As there were no genotype effects for either alcohol or nicotine 2-bottle choice tests, we did not evaluate the effect of *Grk4* deletion on taste preference.

## Discussion

Despite moderate success in the identification of replicable SUD-related genes, numerous challenges preclude discovery and validation of additional genetic variants and loci, including methodological limitations of genotyping microarrays and genotype imputation, diagnostic and phenotypic heterogeneity, genetic signal divergence across related but distinct behaviors, traits, and measures, and the relative lack of diverse genetic ancestry representation. In addition, the majority of genome-wide significant variants and loci are located in non-coding or regulatory regions of the genome with unclear functional consequence, and the extent of linkage disequilibrium complicates efforts to ascertain the true causal variant(s) and gene(s) within a locus. As such, methods and study designs that can isolate the effects of a specific gene mutation or change in gene product in a tightly controlled experimental environment represent ideal conditions under which to validate the effects of novel candidate genes on a phenotype and clarify the biological mechanisms underlying these associations. One such promising approach uses model organisms to employ back-translation of human GWAS findings for orthologous genes and comparable behavioral phenotypes in non-human species. Identifying the role of novel genes in addiction-relevant behaviors, such as drug consumption and withdrawal, requires genetic manipulation in an animal model that can exhibit the relevant behaviors. Transgenic mice are advantageous as they exhibit a wide variety of complex addiction-related behaviors and are frequently used as pre-clinical models. One example of back-translation using mouse models was a GWAS meta-analysis that identified an intronic variant in *KLB* to be significantly associated with alcohol consumption, and subsequently validated the effects of this gene on alcohol consumption in brain-expressed β-Klotho knockout mice^34^. These findings provide preliminary support for the application of back-translation of human GWAS findings to validate novel gene loci and biological relevance, and test for cross-species convergence for SUD-related traits (for additional discussions on the use of model organisms in the 'post-GWAS' era, see^35,36^).

Based on the results of our large-scale GWAS study^13^, we selected three genes, *HGFAC*, *SLC38A9* and *GRK4*, that were associated with altered ADW or CPD to directly test in pre-clinical mouse models of alcohol and nicotine consumption. ADW and CPD are more amenable to modeling in pre-clinical studies since they are consummatory behaviors that can be measured in mice. We used 2-bottle choice tests in which the mice voluntarily consume alcohol or nicotine in their home cage over several weeks. These tests are widely used in the pre-clinical setting to assess voluntary alcohol and nicotine consumption in mice, particularly in the high drug-consuming C57BL/6 mouse strain^24,28,32,33,37–42^. Mice voluntarily consume physiologically relevant levels of drugs as they can exhibit withdrawal symptoms after the drug bottles are withheld^22,29,43,44^. Additionally, these tests do not require animal training, nor food or fluid restriction at any time. All three of the transgenic lines were maintained on the C57BL/6 background, which is the most frequently used for genetic deletion studies and behavioral assessments^40,42,45,46^. Within the C57BL/6 strain, female mice are known to consume more alcohol and nicotine compared with male mice^22,25,29–33^. All three transgenic lines also showed this sex difference in alcohol and nicotine intake, with WT female mice consuming more alcohol and nicotine compared with WT littermate male mice. Thus, we examined the sexes separately within each transgenic line as in our prior work^22,24,28,31^.

We examined transgenic mouse lines with constitutive, global gene deletions (*Hgfac* and *Grk4* KO) or genes with severely impacted function (*Slc39a8* hypomorph HET). Assessing genetic KO mice evaluates the role of the target transcript and protein in these behaviors, which may not be the biological mechanism underlying the association observed in the GWAS. However, a gene deletion may maximize the probability of detecting a genotype effect on alcohol and nicotine consumption in pre-clinical models. Generating a mouse transgenic line that harbors the target variant is a more translational approach and allows for direct testing of the deleteriousness of the variant in addition to behavioral phenotyping; however, this is offset by the time and cost involved in generating the transgenic mice and may not be amenable to testing large numbers of target genes. In addition, transgenic lines with target gene deletions may be readily available if the gene is of interest in research fields other than SUD. Additional limitations of using constitutive, global genetic KO mice are the regulatory processes impacting gene expression are not directly tested and the role of these genes in specific tissues cannot be assessed. Nevertheless, pre-clinical back-translation approaches provide a valuable starting point for investigating the role of novel genes and proteins in AUD and/or TUD biology.

The SNP in the *HGFAC* gene (rs3748034) was predicted to generate a nonsynonymous missense mutation, which was associated with lower levels of ADW in the GWAS^13^. *HGFAC* encodes for hepatocyte growth factor activator and its primary function is as a liver-secreted serum proteinase that converts hepatocyte growth factor inactive precursor (proHGF) to active hepatocyte growth factor (HGF). *HGFAC* dysfunction has been implicated in impaired tissue injury and repair, impaired gastrointestinal function, inflammation, fibrosis and cancer^16^. Low levels of *HGFAC* mRNA have been found in extrahepatic organs including the gastrointestinal tract, kidneys, lungs and central nervous system^16,47^. *Hgfac* KO mice are viable and do not show overt abnormalities or tumorigenesis after 1 year of age^48^. We found reduced alcohol consumption at 10% and 14% alcohol concentrations, and an overall reduction in alcohol preference in the female *Hgfac* KO mice compared with WT littermates. A change in both the consumption and preference increases the likelihood that *Hgfac* deletion produces a meaningful change in alcohol intake in the female mice. No changes in alcohol consumption or preference were observed in the male *Hgfac* KO mice. As the GWAS data on *HGFAC* was not stratified by sex^13^, these data suggest an intriguing potential sex difference in the role of *Hgfac* in alcohol consumption that should be further explored.

Although the mutation in *HGFAC* was not associated with CPD, we tested nicotine consumption and preference in drug naïve *Hgfac* WT and KO mice since alcohol and nicotine addiction mechanisms are known to share genetic factors^7–10^. Interestingly, we found a reduction in nicotine consumption in the male *Hgfac* KO mice at the 100 μg/mL concentration, and an overall reduction in preference for nicotine compared with WT littermates. This reduction in nicotine consumption and preference was not observed for the female *Hgfac* KO mice, again illustrating an intriguing sex difference in alcohol and nicotine responses.

As alcohol consumption is influenced by sweet taste responses^49,50^ and nicotine has a bitter taste^40,51^, we measured preference for saccharin (a non-caloric sweetener) and quinine (a bitter substance) in male and female *Hgfac* KO and WT mice and saw no genotype differences in preference for either sweet or bitter solutions. Thus, the change in alcohol consumption and preference in the female *Hgfac* KO mice, and change in nicotine consumption and preference in the male *Hgfac* KO mice are not confounded by altered sweet and bitter taste perception.

Based on the GWAS findings^13^, these 2-bottle choice tests supported our hypothesis that *Hgfac* was involved in alcohol intake and also provided unexpected data implicating *Hgfac* in nicotine intake. Thus, *Hgfac* may be another common molecular mechanism that is involved in both alcohol and nicotine addiction, and its involvement appears to differ by sex. A recent study examining SUD-related gene interactions found sex differences in the pattern of genetic interactions between *PPP1R12B*, a member of the DARPP-32 signaling family important for neurotransmission, and *HGFAC*^52^*.* The meta-analysis identified *PPP1R12B* as an interacting gene with *HGFAC* in human females but not in males^52^. In addition to genetic studies, studies using different pre-clinical models will be required to better understand what other aspects of addiction-related behavior are altered in *Hgfac* KO mice. As the changes in alcohol and nicotine consumption each occurred in one sex only, further investigation into the molecular and biological mechanisms will be needed to understand how altered *Hgfac* expression influences alcohol and nicotine addiction biology with a particular emphasis on sex differences in the underlying biological mechanisms.

The SNP in *SLC39A8* (rs13107325) was predicted to generate a nonsynonymous missense mutation, which was associated with lower levels of ADW in the GWAS^13^. This SNP is highly pleiotropic and has been associated with schizophrenia, Crohn’s disease, serum manganese and body mass index and others^53^. *Slc39a8* has not been studied in the context of alcohol or nicotine consumption phenotypes in pre-clinical models. *Slc39a8* encodes for a Zrt- and Irt-like protein 8 (ZIP8) which functions as a zinc transporter^17,18^. ZIP8 is expressed widely expressed in the body, with higher levels in the lung, testis, kidney, liver and lower levels in the brain, heart, intestines and pancreas in adult mice^54–56^. The *Slc39a8* hypomorph allele consists of a retained neomycin cassette that results in ~ 5–8% of normal mRNA expression levels^20^. Homozygous *Slc39a8*^neo/neo^ mice are not viable, thus we used *Slc39a8*^+/neo^ HET mice in our experiments. Adult *Slc39a8* HET mice exhibit increased incidence of spontaneous liver neoplastic nodules between 13 and 21 months of age^57^—we performed all our experiments in mice well under the age of 13 months. Although the SNP in *SLC39A8* was associated with a decrease in ADW, we did not find genotype differences in alcohol consumption or preference in male or female *Slc39a8* WT and HET mice. Furthermore, we found no differences in nicotine consumption or preference in between the female *Slc39a8* WT and HET mice. Male *Slc39a8* HET mice showed slightly greater overall nicotine preference, with no changes in nicotine consumption, compared with WT mice. The increased nicotine preference in male *Slc39a8* HET mice cannot be explained by differences in total fluid consumption, as *Slc39a8* HET mice consumed less fluid per body weight compared with WT littermates throughout the experiment, and male *Slc39a8* HET mice did not show differences in body weight compared with WT littermates. Further experiments examining nicotine conditioned place preference or operant nicotine responding in male mice will be useful in understanding the role of *Slc39a8* in nicotine-related phenotypes.

The SNP in *GRK4* (rs1024323) was predicted to generate a nonsynonymous missense mutation, which was associated with lower levels of CPD in the GWAS^13^. *GRK4* encodes for G-protein coupled receptor kinase 4, which contributes to the regulation of G protein coupled receptor (GPCR) activity. After activation of a GPCR, GRK proteins phosphorylate the receptor blocking further receptor activation and recruiting cellular processes that internalize the GPCR. *Grk4* transcript is expressed in the kidney, bone, heart, testes, intestine and brain^19,58,59^. *Grk4* has primarily been implicated in hypertension^19,60^. Although the SNP in *Grk4* was associated with a decrease in CPD, we found no difference in nicotine consumption and preference in *Grk4* KO mice compared with WT littermates in either sex. In addition, there was no effect of *Grk4* deletion in alcohol consumption or preference in either sex.

Of the three genes tested in this study, we found altered drug consumption and preference in *Hgfac* that supported our hypotheses from the GWAS data. Although we did not find a genotype effect in the *Slc39a8* and *Grk4* transgenic lines in these chronic 2-bottle choice tests, it is possible that *Slc39a8* and *Grk4* influence alcohol and nicotine addiction behaviors and mechanisms that are not captured by this procedure. As with all pre-clinical models, chronic 2-bottle choice tests assess only a small aspect of alcohol and nicotine addiction-related behaviors. Indeed, variations of the alcohol 2-bottle choice test have been implemented to capture different aspects of alcohol intake compared with continuous chronic access, such as the binge drinking-in-the-dark procedure^61^ and models that incorporate repeated cycles of abstinence^29^. In addition, examining the impact of these genes on alcohol and nicotine metabolism can provide further insight into potential mechanisms of action. Identifying additional behavioral models and tests will be important in future studies of the impact of these genes in AUD and/or TUD biology.

## Data Availability

The datasets generated during and/or analyzed during the current study are available from the corresponding author on request.
